# Incidence of sexually transmitted infections during pregnancy

**DOI:** 10.1371/journal.pone.0197696

**Published:** 2018-05-24

**Authors:** Chloe A. Teasdale, Elaine J. Abrams, Mary Ann Chiasson, Jessica Justman, Kelly Blanchard, Heidi E. Jones

**Affiliations:** 1 ICAP, Mailman School of Public Health, Columbia University, New York, NY, United States of America; 2 Department of Epidemiology, Mailman School of Public Health, Columbia University, New York, NY, United States of America; 3 Ibis Reproductive Health, Cambridge, MA, United States of America; 4 Department of Epidemiology, City University of New York School of Public Health, New York, NY, United States of America; London School of Hygiene and Tropical Medicine Department of Population Health, UNITED KINGDOM

## Abstract

Prevalence of sexually transmitted infections (STI) is high among pregnant women in certain settings. We estimated STI incidence and compared STI risk in pregnant and non-pregnant women. Data came from the Methods for Improving Reproductive Health in Africa (MIRA) study conducted in South Africa and Zimbabwe 2003–2006. Women aged 18–50 years with at least one follow-up visit within 6 months of enrollment were included. Follow-up visits included laboratory testing for pregnancy, chlamydia, gonorrhea, trichomoniasis, and HIV, as well as self-report of hormonal contraceptive (HC) use, sexual behaviors and intravaginal practices. All visits were classified according to pregnancy status. Incidence of each STI was calculated using follow-up time. Cox proportional hazards models were fitted using pregnancy as a time-varying exposure and sexual behaviors and intravaginal practices as time-varying covariates. Among 4,549 women, 766 (16.8%) had a positive pregnancy test. Median follow-up time was 18 months [IQR: 12–24]. The overall incidence rate of chlamydia was 6.7 per 100 person years (py) and 9.9/100py during pregnancy; gonorrhea incidence was 2.7/100py and 4.9/100py during pregnancy; trichomoniasis incidence was 7.1/100py overall and 9.2/100py during pregnancy. Overall HIV incidence was 3.9/100py and 3.8/100py during pregnancy. In crude models, pregnancy increased risk for chlamydia (hazard ratio (HR) 1.5, 95%CI: 1.1–1.2), however there was no increased risk of any measured STI in adjusted models. STI Incidence was high during pregnancy however pregnancy did not increase STI risk after adjustment for sexual behaviors. Greater efforts are needed to help pregnant women avoid STIs.

## Introduction

High prevalence of sexually transmitted infections (STI) has been observed in some populations of pregnant women. Data from routine screening in antenatal care settings in sub-Saharan Africa have shown prevalence of common STIs, including chlamydia, gonorrhea and syphilis, to be as high as 15% [1, 2].^.^ Almost half of all women under 30 years of age are infected with Herpes Simplex Virus type 2 (HSV-2] across sub-Saharan Africa [3] and up to 30% of pregnant women have HIV in southern Africa [4]. Pregnant adolescents in other parts of the world have also been found to have high STI prevalence; in Portugal one study found 11% of pregnant teenagers to be infected with trichomoniasis and 5% with gonorrhea [5].

STIs can cause chronic pain as well as permanent infertility in women who do not receive treatment and are of particular concern during pregnancy as they are associated with adverse outcomes including stillbirth and low birth weight and some can also be transmitted to infants leading to blindness and developmental delays [6, 7]. HIV is the leading cause of maternal mortality in high prevalence settings, increasing the risk of death six-fold for pregnant women and new mothers [8]. Mother-to-child transmission (MTCT) of HIV and HSV-2 results in lifelong infection and HIV is associated with high mortality in untreated infected children [9, 10]. Incident maternal infection with HIV and HSV-2 during pregnancy may confer higher risk of MTCT [10, 11].

While a small number of studies have shown that incidence of common curable STIs, as well as HIV, continues during pregnancy, few have evaluated whether pregnancy increases the risk of STI acquisition [12–16]. Studies have compared incidence in pregnant and non-pregnant women only for HPV and HIV showing no increased risk of HPV during pregnancy [13] but conflicting results for HIV [17–19]. Data indicate that pregnant women tend to report less condom use compared to women who are not pregnant which could increase STI incidence [20, 21]. In addition to sexual risk behaviors, intravaginal practices, including douching, cleaning, wiping and insertion of materials and substances into the vagina, have also been studied as potential risk factors for STI acquisition, particularly HIV [22], and may vary during pregnancy [21]. Alternations in immune function [23–25] and physiological changes to the cervix that occur during pregnancy are thought to be potential biological pathways of increased risk [26–29].

In light of the significant consequences of STIs for women and their children, it is critical to investigate whether pregnancy increases a woman’s risk of STI acquisition. In this analysis, we measured incidence of four common STIs (chlamydia, gonorrhea, trichomoniasis and HIV) among pregnant and non-pregnant women and compared STI risk based on pregnancy status using data from a randomized clinical trial conducted in South Africa and Zimbabwe.

## Methods

Data came from the Methods for Improved Reproductive Health in Africa (MIRA) study, an open-label trial of the diaphragm and lubricant gel for prevention of HIV infection in women which was approved by institutional review boards (IRB) in the United States, South Africa and Zimbabwe (ClinicalTrials.gov: NCT00121459). The MIRA trial enrolled HIV-negative women >18 years of age in South Africa and Zimbabwe. During 12–24 months of study follow-up, participants attended quarterly visits which included STI/HIV testing, and risk reduction counseling including unlimited condoms. Participants self-reported sexual activity, contraceptive and condom use, and intravaginal practices through clinician-administered interviews and audio computer-assisted self-interviewing (ACASI). Women who became pregnant continued in the study during and after pregnancy (women in the diaphragm arm had the option to discontinue use). All women testing positive for HIV were referred for treatment; treatment for curable STIs was given at study sites. Data were not available from care women may have received at non-study health facilities.

For this analysis, we included HIV-negative women 18–50 years of age, including those who tested positive and received treatment for chlamydia, gonorrhea, trichomoniasis and syphilis at enrollment. Women were excluded from the analysis if their first study visit was more than six months after the enrollment date and if they tested positive for chlamydia, gonorrhea or trichomoniasis at the two week visit (indicating incomplete treatment or treatment failure at enrollment). Data from study visits that occurred more than six weeks after a woman gave birth were excluded as incidence of STIs during the post-partum period may differ from other non-pregnant periods [30]. In addition, visits with greater than a six month gap were censored at the last visit prior to the gap, as were visits that occurred after an HIV diagnosis as behavior may change following diagnosis.

Pregnancy status was ascertained through urine testing at all scheduled visits (pregnancy testing was not conducted when women attended non-quarterly visits to receive care for STI symptoms). Women were considered pregnant if they had a positive human chorionic gonadotropin (HCG) urine pregnancy test and non-pregnant at all visits with negative pregnancy tests even if they reported pregnancies between visits resulting in miscarriage or termination. Pregnancy status at a given visit determined exposure status during the interval of time prior to that visit (if a woman was pregnant at her third quarterly visit, she was considered pregnant for the full interval between the second and third visits). Women did not report last menstrual period and ultrasound testing was not used to determine gestational age; women were considered to be pregnant for the entire interval for the visit when they became pregnant. Visits within six weeks of a birth were considered pregnant because women were pregnant for most of the visit interval. Given the potential for hormonal contraceptives (HC) to increase risk of STI/HIV [31, 32], non-pregnant visits were further classified into three groups according to self-reported HC use: oral contraceptives (combined estrogen and progesterone and progesterone-only pills), injectable HC (Net-En and Depo Provera), and no use of HC including report of condoms, diaphragm and natural methods or no method. For women with two pregnancies during follow-up, only the first pregnancy was included in the analysis.

Four STI outcomes were examined: chlamydia, gonorrhea, trichomoniasis and HIV. DNA polymerase chain reaction (PCR) testing of urine was used to diagnosis chlamydia, gonorrhea and trichomoniasis (Roche COBAS® AMPLICOR, Branchburg, NJ, USA). Two rapid HIV tests were conducted on whole blood samples with Determine HIV-1/2 (Abbot Laboratories, Tokyo, Japan) or Oraquick (Orasure Technologies, Bethlehem, PA, USA). Discordant rapid tests were confirmed with enzyme-linked immunosorbent assay (ELISA). Testing laboratories in Zimbabwe and South Africa were monitored for quality control during the study. As previous data have shown that PCR testing of urine may have lower sensitivity and specificity for detecting chlamydia, gonorrhea and trichomoniasis (compared to vaginal swabs), we report both the observed and adjusted prevalence estimates [33] at enrollment for the three infections using estimated sensitivity and specificity estimates for urine testing [34, 35].

We estimated time to first incident infection of each of the four STIs (incident infection defined as the first positive test after a previous negative test). STI infection outcomes were assigned to the quarterly study visit with positive test result and matched with the behavioral data reported at the same visit corresponding to the period *prior* to the visit. Positive STI test results from non-quarterly visits (when women sought additional services generally due to symptoms) were carried forward to the subsequent quarterly study visit and matched with the corresponding behavior data from the same visit interval.

Hypothesized confounders included age, education, intervention arm (diaphragm or control) and contraceptive use at enrollment, along with a variable indicating any report at enrollment of high risk sexual behaviors including anal sex, sex in exchange for drugs or money, two or more sexual partners, new sexual partner or sex under the influence of drugs or alcohol. Women also reported male partners risk behaviors at enrollment with data summarized into a high risk variable indicating any HIV-infected status, away from home >one month in past year, use of drugs during sex or suspicion or knowledge of other sex partners. Behaviors reported by women at all quarterly study visits included frequency of sexual activity, condom use, sex in exchange for money or drugs, two or more sex partners, new sex partner and suspecting or knowing that a male partner had other partners. Intravaginal practices were categorized into three groups: washing, wiping, or inserting paper products into the vagina (not for menstruation).

Descriptive statistics were used to compare demographic characteristics and frequency of reported sexual behaviors and intravaginal practices at enrollment for women with and without positive pregnancy tests. To estimate incidence of STIs during pregnant and non-pregnant periods, each of the four STIs was examined separately with follow-up time for participants calculated from enrollment date to the first diagnosis of each STI or end of follow-up. For intervals between visits culminating in incident STI infection, the midpoint between visits with the last negative and first positive test was used. Follow-up time at risk was estimated by summing the intervals of time between visits for each participant based on four exposure groups assigned to each visit interval: pregnant, non-pregnant/oral HC, non-pregnant/injectable HC and non-pregnant/no HC use. Incidence rates in person years were calculated by dividing the corresponding follow-up time at risk for each exposure group separately as well as overall.

Bivariable and multivariable Cox proportional hazards models were fitted to examine pregnancy status and incidence of the four STI separately and a composite endpoint of first incidence of any of four infections. Adjusted models included a priori hypothesized confounders. Pregnancy was treated as a time-varying exposure and sexual risk behaviors and vaginal practices were treated as time-varying confounders; demographic and enrollment characteristics were modeled as time-fixed confounders. Study location was tested for effect measure modification using a cross-product term of location and pregnancy status.

## Results

Of the 4,948 women analyzed in the MIRA trial, 4,935 (99.7%) were 18–50 years of age at enrollment and among those, 4,553 (92.3%) had at least one follow-up visit with STI and pregnancy testing within six months of the date of enrollment (four women were excluded as a result of incomplete treatment for curable STIs at enrollment). Among 4,549 women included in the analysis, 766 (16.8%) had a positive pregnancy test during follow-up (35 women reported a termination, 83 women reported miscarriages and one woman reported an ectopic pregnancy). Of the 24,337 visits included in the analysis, 1,609 (6.6%) occurred during a pregnancy. Median follow-up time was 15 months [IQR: 12–21] for women with pregnancies and 18 months [IQR: 12–24] for women without a pregnancy (p = 0.47).

At enrollment, women who became pregnant during follow-up were more likely to be from Zimbabwe and were younger; median age for women with pregnancies was 25 years (interquartile range (IQR): 21–30) whereas median age of women without pregnancies was 28 years (IQR: 23–35) (p<0.0001) (Table 1). Compared to women who did not have pregnancies, women with pregnancies were more likely to have completed high school and were less like to have children at enrollment; they were also more likely to report use of oral contraceptives, to have had two or more sexual partners at enrollment and to be infected with HSV-2 (Table 1). There were no other differences in demographics or reported sexual behaviors, male partner characteristics or STI status.

**Table 1:** Characteristics at enrollment of 4,549 MIRA participants with and without pregnancies during follow-up.

Characteristics at enrollment	Total			Women with no pregnancies		Women with lab confirmed pregnancies	
	N		%	N	%	N	%
	4549		100.0%	3783	83.2%	766	16.8%
Study location							
Harare	2255		49.6%	1847	48.9%	408^a^	53.3%
Durban	1372		30.2%	1173	30.9%	199	26.0%
Johannesburg	922		20.3%	763	20.2%	159	20.8%
Age							
Median age (IQR)	27 (22–34)			28 (23–35)		25^b^ (21–30)	
18–24	1725		37.9%	1342	35.5%	383	50.0%
25–34	1782		39.2%	1472	38.9%	310	40.5%
≥35	1042		22.9%	969	25.6%	73	9.5%
Completed high school (11 yrs school)	2347		51.6%	1904	50.3%	443^c^	57.8%
Paid employment	1039		22.4%	883	23.4%	156	20.4%
Married	2695		59.3%	2245	59.4%	450	58.8%
Lives with male partner	3081		67.7%	2572	68.0%	509	66.5%
Number or previous pregnancies							
None	387		8.5%	280	7.4%	107^b^	14.0%
1 previous pregnancy	1446		31.8%	1151	30.4%	295	38.5%
2+ previous pregnancies	2716		59.7%	2352	62.2%	364	47.5%
Current birth control (not mutually exclusive)							
Combined pills	991		21.8%	770	20.4%	221^b^	28.9%
Injectable hormones	1129		24.8%	1029	27.2%	100^b^	13.1%
Progesterone only pills	663		14.6%	566	15.0%	97	12.7%
Intrauterine device	16		0.4%	15	0.4%	1	0.1%
Condoms (male or female) (only)	1723		37.9%	1419	37.5%	304	39.7%
None (natural, withdrawal, tradition)	247		5.4%	194	5.1%	53	6.9%
Regular sexual partner	1852		40.7%	1537	40.6%	315	41.1%
Age at first sex, mean (IQR)	18 (16–19)			18 (16–19)		18 (16–19)	
Sex acts per week							
None	1877		41.3%	1551	41.0%	326	42.6%
1–2 per week	275		6.1%	227	6.0%	48	6.3%
≥3 per week	2397		52.7%	2005	53.0%	392	51.2%
Number of sexual partners in past 3 months							
None	273		6.0%	249	6.6%	24^b^	3.1%
1 partner	3877		85.5%	3222	85.4%	655	85.5%
2+	387		8.5%	300	8.0%	87.0	11.4%
Condom use (male or female) at last sex	3200		70.4%	2653	70.1%	547	71.4%
Condom use (vaginal sex) in last three months							
Never	1338		29.5%	1132	30.0%	206	26.9%
Sometimes	1767		38.9%	1449	38.4%	318	41.5%
Every time	1433		31.6%	1191	31.6%	242	31.6%
Exchange for money or drugs past 3 months	360		7.9%	303	8.0%	57	7.4%
Anal sex (ever)	669		14.8%	53	14.7%	116	15.1%
Sex while intoxicated	168		26.7%	136	26.8%	32	26.0%
High risk	1059		23.3%	874	23.1%	185	24.2%
(>2 partners, exchange, anal, sex w/ drugs/alcohol)							
Partner age							
Same age (+/-5 years)	2510		57.8%	2077	57.7%	433	58.2%
Younger (more than 5 years)	50		1.2%	47	1.3%	3	0.4%
Older (more than 5 years)	1785		41.1%	1477	41.0%	308	41.4%
Partner tested HIV positive							
Yes	156		3.4%	135	3.6%	21	2.7%
No/don't know	4380		96.6%	3635	96.4%	745	97.3%
Partner away from home >2 months	490		10.9%	413	10.9%	77	10.1%
Partner circumcised	976		21.5%	805	21.4%	171	22.3%
Suspects/knows partner concurrency	1392		30.7%	1159	30.7%	233	30.4%
Physical or verbal abuse	821		33.0%	693	33.0%	128	33.4%
Partner used force to get sex	244		9.8%	208	9.9%	36	9.4%
Sex w/ partner with drugs or alcohol	1622		35.9%	1351	36.0%	271	35.5%
Partner high risk	2836		62.3%	2363	62.5%	473	61.8%
(HIV+, away >1 month, other partners, drugs)							
Vaginal washing	3756		82.6%	3119	82.5%	637	83.2%
Vaginal wiping	2560		56.3%	2117	56.0%	443	57.8%
Vaginal insertion (non-menstruation related)	946		20.8%	790	20.9%	156	20.4%
Diaphragm arm	2273		50.0%	1890	50.0%	383	50.0%
Positive for STI (enrollment)							
Chlamydia	195		4.3%	155	4.1%	40	5.2%
Corrected chlamydia prevalence estimate			4.6%		4.3%		5.7%
Gonorrhea	34		0.8%	29	0.8%	5	0.7%
Corrected chlamydia prevalence estimate			0.9%		0.9%		1.1%
Trichomoniasis	166		3.7%	133	3.5%	33	4.3%
Corrected chlamydia prevalence estimate			3.1%		2.8%		4.0%
HSV-2	2656		58.4%	2254	59.6%	402^c^	52.5%

^a^p<0.05

^b^p<0.01

^**c**^p<0.0001

Overall incidence of chlamydia was 6.7 per 100 person years (py) and was highest when women were using injectable HC (10.1/100py) (Table 2). Gonorrhea and trichomoniasis incidence overall were 2.7/100py and 7.1/100py, respectively, and both were highest during periods when women were pregnant; 4.9/100py and 9.2/100py. The rate of HIV incidence overall was 3.9/100py with highest incidence occurring when women reported injectable HC (4.8/100py) (Table 2).

**Table 2:** Incidence rates of four STIs by pregnancy and hormonal contraceptive exposure group.

	Overall			Pregnant visits			Non-pregnant & oral HC use visits			Non-pregnant & injectable HC use visits			Non-pregnant & no HC use		
	Cases	Person years	IR p/Pty	Cases	Person years	IR p/yr	Cases	Person years	IR p/yr	Cases	Person years	IR p/yr	Cases	Person years	IR p/yr
Chlamydia	400	5935	**6.7**	38	383	**9.9**	84	2066	**4.1**	146	1440	**10.1**	132	2045	**6.5**
Gonorrhea	165	6130	**2.7**	20	406	**4.9**	34	2114	**1.6**	49	1508	**3.2**	62	2103	**2.9**
Trichomoniasis	420	5935	**7.1**	36	390	**9.2**	115	2040	**5.6**	109	1469	**7.4**	160	2036	**7.9**
HIV	240	6199	**3.9**	16	417	**3.8**	56	2127	**2.6**	74	1530	**4.8**	94	2124	**4.4**

In unadjusted models, pregnancy was associated with an increased risk of chlamydia compared to non-pregnant periods with no HC use (hazard ratio (HR) 1.5; 95%CI 1.1–2.2) (Table 3). In multivariable adjusted models however, the effect of pregnancy on chlamydia incidence was attenuated and no longer significant. Pregnancy also appeared to increase the hazards of gonorrhea, trichomoniasis and the combined endpoint of any STI however these findings did not reach the threshold for significance. Pregnancy did not appear to increase the risk of HIV infection (Table 4). Neither oral nor injectable HC use was associated with increased hazards of any of the four STIs. Site location was not found to be an effect modifier of the relationship between pregnancy and STI risk in adjusted models.

**Table 3:** Crude and adjusted^a^ hazard ratios estimating the relationship between pregnancy status and incident STIs (N = 4,549).

	Chlamydia				Gonorrhea					Trichomoniasis					
	HR	95%CI	AHR^a^	95%CI	HR	95%CI	AHR^a^		95%CI	HR	95%CI		AHR^a^		95%CI
**Pregnancy exposure group** (ref: not pregnant/noHC)															
Pregnant	**1.52**	**1.06–2.18**^**b**^	1.29	0.89–1.88	1.59	0.96–2.63		1.52	0.89–2.57	1.13	0.79–1.62			1.27	0.86–1.86
Not pregnant/oral HC	**0.65**	**0.49–0.85**^**c**^	0.92	0.63–1.33	0.55	0.36–0.84		0.83	0.47–1.47	**0.72**	**0.57–0.91**^**b**^			0.92	0.65–1.29
Not pregnant/injectable HC	**1.57**	**1.24–1.98**^**c**^	1.32	0.99–1.76	1.09	0.75–1.59		0.94	0.60–1.47	0.94	0.74–1.20			0.99	0.73–1.40
Study location (ref: Harare, Zimbabwe)															
Johannesburg, South Africa	**2.73**	**2.15–3.47**^**d**^	**2.33**	**1.71–3.19**^**d**^	**2.89**	**2.00–4.16**^**d**^		**2.19**	**1.34–3.59**^**c**^	**1.74**	**1.40–2.15**^**d**^			**1.51**	**1.14–1.99**^**c**^
Durban, South Africa	**2.88**	**2.32–3.74**^**d**^	**2.22**	**1.57–3.14**^**d**^	**2.58**	**1.71–3.90**^**d**^		**1.79**	**1.03–3.10**^**c**^	1.28	0.98–1.67			1.11	0.80–1.57
Age (ref: ≥35)															
18–24 years	**4.95**	**3.46–7.09**^**d**^	**5.49**	**3.74–8.06**^**d**^	**4.17**	**2.46–7.06**^**d**^		**5.31**	**3.01–9.36**^**d**^	0.96	0.76–1.22			1.23	0.94–1.62
25–34 years	**2.09**	**1.43–3.06**^**c**^	**2.41**	**1.62–3.58**^**d**^	**1.79**	**1.02–3.15**^**b**^		**2.27**	**1.26–4.01**^**b**^	**0.73**	**0.57–0.93**^**b**^			0.89	0.68–1.17
Completed high school	1.45	1.19–1.77	1.09	0.88–1.36	1.30	0.96–1.77		0.97	0.70–1.35	**0.74**	**0.61–0.90**^**c**^			0.82	0.66–1.01
Diaphragm arm	1.20	0.99–1.46	1.16	0.94–1.44	0.94	0.69–1.28		1.03	0.75–1.43	0.92	0.76–1.12			0.93	0.75–1.14
Contraceptive use at enrollment (ref: none)															
Oral contraceptives	**0.65**	**0.45–0.95**^**b**^	1.03	0.65–1.63	0.89	0.47–1.70		1.75	0.81–3.76	**0.52**	**0.39–0.70**^**d**^			0.81	0.55–1.21
Injectable contraceptives	1.43	1.00–2.05	0.95	0.64–1.42	1.61	0.86–3.03		1.40	0.71–2.78	**0.66**	**0.49–0.90**^**b**^			0.74	0.52–1.06
Other^e^	1.19	0.83–1.71	1.07	0.74–1.54	1.78	0.96–3.31		1.68	0.90–3.13	**0.56**	**0.41–0.76**^**c**^			0.60	**0.44–0.82**^**c**^
High risk sex at enrollment^f^	1.28	1.03–1.59	1.21	0.97–1.52	0.79	0.53–1.16		0.68	0.46–1.02	1.22	0.98–1.51			1.12	0.89–1.40
Partner circumcised at enrollment	**1.24**	**0.99–1.56**	1.09	0.85–1.39	**1.65**	**1.19–2.31**^**b**^		**1.57**	**1.01–2.25**^**b**^	1.00	0.79–1.27			1.00	0.78–1.29
Partner high risk at enrollment	1.25	1.02–1.54	1.04	0.83–1.29	**1.77**	**1.25–2.51**^**c**^		1.40	0.97–2.00	**1.39**	**1.13–1.71**^**c**^			1.02	0.96–1.48
STI at enrollment ^g^	1.21	0.99–1.49	**1.47**	**1.18–1.82**^**c**^	**1.90**	**1.34–2.70**^**c**^		**2.16**	**1.50–3.11**^**d**^	**1.96**	**1.57–2.44**^**d**^			**1.80**	**1.42–2.27**^**d**^
Vaginal sex since last visit (ref: none)	0.89	0.60–1.35	0.91	0.56–1.48	1.00	0.51–1.96		1.09	0.51–2.34	0.82	0.56–1.22			0.82	0.51–1.30
≥3 Vaginal sex acts per week (ref:0–2)	0.94	0.75–1.16	1.18	0.93–1.51	**0.70**	**0.50–0.96**^**b**^		0.83	0.59–1.19	**0.79**	**0.64–0.98**^**b**^			0.91	0.72–1.16
No condom use at last sex	1.06	0.84–1.33	0.93	0.71–1.21	**0.61**	**0.41–0.92**^**b**^		**0.56**	**0.36–0.89**^**b**^	1.05	0.84–1.32			1.04	0.79–1.36
Unprotected sex since last visit	**1.41**	**1.41–1.74**^**c**^	1.27	0.99–1.63	1.12	0.82–1.54		1.20	0.84–1.71	1.18	0.97–1.44			1.07	0.84–1.36
Sex in exchange for drugs or money	**1.68**	**1.06–2.66**^**b**^	1.30	0.80–2.13	1.50	0.70–3.21		1.36	0.61–3.06	**1.61**	**1.02–2.55**^**b**^			1.16	0.71–1.88
2 or more sexual partners	**2.89**	**2.22–3.77**^**d**^	**1.55**	**1.13–2.14**^**c**^	**2.97**	**1.98–4.47**^**d**^		1.36	0.83–2.23	**2.13**	**1.60–2.84**^**d**^			**1.77**	**1.26–2.49**^**c**^
New sexual partner	**1.85**	**1.44–2.37**^**d**^	1.12	0.85–1.49	**2.36**	**1.64–3.39**^**d**^		1.48	0.98–2.25	**1.93**	**1.22–2.04**^**c**^			1.11	0.83–1.48
Suspects/knows partner concurrency	**1.65**	**1.34–2.02**^**d**^	1.21	0.97–1.52	**2.42**	**1.78–3.30**^**d**^		**1.70**	**1.21–2.39**^**c**^	**1.50**	**1.22–1.84**^**c**^			1.22	0.98–1.52
Vaginal washing (any reported)	0.98	0.78–1.23	0.96	0.75–1.23	1.09	0.76–1.57		1.13	0.76–1.67	1.01	0.81–1.27			1.03	0.81–1.31
Vaginal wiping (any reported)	0.91	0.74–1.11	0.92	0.73–1.15	0.99	0.72–1.35		1.04	0.73–1.48	1.05	0.86–1.28			0.92	0.74–1.15
Insertion of paper (any reported)	1.13	0.86–1.50	1.24	0.92–1.69	0.87	0.54–1.41		0.88	0.53–1.50	**1.36**	**1.05–1.76**^**e**^			1.27	0.96–1.69

^a^Multivariable models were adjusted for all covariates listed in the table

^b^p<0.05

^c^p<0.01

^**d**^p<0.0001

^e^Other (diaphragm, IUD, condoms, vasectomy)

^f^High risk sex: exchange, anal sex, >2 partners, new partner, new regular partner

^g^STI at enrollment: chlamydia, gonorrhea, trichomoniasis, HSV-2

**Table 4:** Crude and adjusted^a^ hazard ratios estimating the relationship between pregnancy status and incident STIs (N = 4,549).

	HIV				Combined STI endpoint			
	HR	95%CI	AHR^a^	95%CI	HR	95%CI	AHR^a^	95%CI
**Pregnancy exposure group** (ref: not pregnant/no HC)								
Pregnant	0.78	046–1.33	0.75	0.43–1.30	1.18	0.93–1.51	1.15	0.89–1.49
Not pregnant/oral HC	**0.58**	**0.42–0.81**^**c**^	0.82	0.52–1.31	**0.66**	**0.66–0.78**^**d**^	0.84	0.67–1.06
Not pregnant/injectable HC	1.07	0.8–1.5	0.81	0.56–1.18	1.13	0.97–1.32	1.07	0.88–1.29
Study location (ref: Harare, Zimbabwe)								
Johannesburg, South Africa	**2.66**	**2.01–3.53**^**b**^	**1.91**	**1.31–2.77**^**c**^	2.13	**1.5–2.47**^**d**^	1.80	**1.49–2.17**^**d**^
Durban, South Africa	1.30	0.88–1.91	0.89	0.56–1.44	1.80	**1.52–2.13**^**d**^	1.46	**1.12–1.82**^**c**^
Age (ref: ≥35)								
18–24 years	**2.09**	**1.44–3.02**^**b**^	**2.51**	**1.67–3.78**^**d**^	**1.84**	**1.55–2.19**^**d**^	**2.27**	**1.87–2.76**^**d**^
25–34 years	1.34	0.91–1.97	**1.65**	**1.01–2.49**^**b**^	1.06	0.89–1.27	1.27	**1.05–1.56**^**b**^
Completed high school	1.18	0.91–1.53	1.18	0.90–1.56	1.06	0.94–1.21	1.00	0.87–1.14
Diaphragm arm (ref: control)	1.00	0.80–1.29	0.99	0.76–1.31	1.00	0.88–1.14	1.01	0.88–1.16
Contraceptive use, enrollment (ref: none)								
Oral contraceptives	0.85	0.50–1.43	1.35	0.72–2.54	**0.64**	**0.52–0.80**^**c**^	1.05	0.79–1.39
Injectable contraceptives	**1.79**	**1.07–2.97**^**b**^	**1.83**	**1.05–3.18**^**b**^	1.00	0.81–1.26	0.91	0.70–1.17
Other ^e^	1.43	0.85–2.39	1.49	0.89–2.49	0.91	0.73–1.14	0.92	0.73–1.15
High risk sex at enrollment ^f^	1.09	0.82–1.46	1.01	0.81–1.48	1.08	0.93–1.25	0.99	0.85–1.16
Partner circumcised at enrollment	0.94	0.68–1.29	1.00	0.71–1.40	1.43	0.98–1.33	1.08	0.92–1.27
Partner high risk at enrollment	**1.61**	**1.21–2.13**^**c**^	1.29	0.96–1.74	**1.36**	**1.19–1.56**^**d**^	**1.16**	**1.00–1.33**^**b**^
STI at enrollment ^g^	**2.00**	**1.49–2.69**^**d**^	**2.09**	**1.54–2.85**^**d**^	**1.65**	**1.43–1.89**^**d**^	**1.74**	**1.50–2.01**^**d**^
Vaginal sex since last visit (ref: none)	1.06	0.59–1.89	1.00	0.51–1.92	0.87	0.67–1.14	0.93	0.68–1.27
≥3 Vaginal sex acts per week (ref:0–2)	0.77	0.59–1.02	0.86	0.64–1.16	0.82	**0.71–0.94**^**c**^	0.96	0.82–1.12
No condom use at last sex	1.00	0.74–1.34	0.94	0.66–1.34	0.99	0.85–1.15	0.92	0.77–1.10
Unprotected sex since last visit	1.26	0.97–1.64	1.22	0.89–1.66	**1.25**	**1.09–1.42**^**c**^	**1.19**	**1.02–1.39**^**b**^
Sex in exchange for drugs or money	1.23	0.61–2.49	0.90	0.43–1.89	**1.58**	**1.17–2.16**^**c**^	1.31	0.94–1.81
2 or more sexual partners	**2.29**	**1.58–3.33**^**d**^	1.45	0.93–2.25	**2.29**	**1.90–2.76**^**d**^	**1.48**	**1.19–1.85**^**b**^
New sexual partner	**1.94**	**1.40–2.68**^**d**^	1.25	0.87–1.80	**1.71**	**1.45–2.02**^**d**^	1.17	0.97–1.40
Suspects/knows partner concurrency	**1.96**	**1.51–2.55**^**d**^	**1.42**	**1.07–1.88**^**b**^	**1.62**	**1.41–1.85**^**d**^	**1.26**	**1.09–1.46**^**c**^
Vaginal washing (any reported)	1.06	0.78–1.42	0.94	0.68–1.30	0.99	0.86–1.15	0.98	0.83–1.15
Vaginal wiping (any reported)	**1.37**	**1.06–1.78**^**b**^	**1.41**	**1.05–1.89**^**b**^	1.02	0.90–1.17	1.00	0.86–1.16
Insertion of paper products (any reported)	1.27	0.90–1.81	1.12	0.77–1.64	1.15	0.97–1.38	1.13	0.93–1.37

^a^Multivariable models were adjusted for all covariates listed in the table

^b^p<0.05

^c^p<0.01

^**d**^p<0.0001

^e^Other (diaphragm, IUD, condoms, vasectomy)

^f^High risk sex: exchange, anal sex, >2 partners, new partner, new regular partner

^g^STI at enrollment: chlamydia, gonorrhea, trichomoniasis, HSV-2

## Discussion

In this secondary analysis of data from an RCT conducted in South Africa and Zimbabwe, we examined the association between pregnancy status and incidence of chlamydia, gonorrhea, trichomoniasis and HIV. The analysis included 4,549 women 18–50 years of age of whom, 766 (16.8%) had a pregnancy during follow-up. We found that hazard rates of chlamydia, gonorrhea and to a less extent trichomoniasis were higher during periods when women were pregnant, however pregnancy did not appear to increase the hazards of STI in multivariable models adjusted for demographic and time-varying self-reported behavioral risk factors and vaginal practices.

Our data showed high incidence of chlamydia, gonorrhea and trichomoniasis during pregnancy and are some of the first reported data on incidence of these infections in pregnant women. Pregnant women in our study had higher HIV incidence (3.8 per 100py) than that reported from two studies conducted in Uganda and Zimbabwe (2004–2007) of between 1.6 [18] and 2.3/100py [19] but lower than data from a study conducted across seven African countries which found incidence of 7.4/100py (2004–2007) [17]. Among non-pregnant women, incidence rates in Southern Africa have been shown to range from 3.0 to 6.5/100py [36–38]. Our data are further confirmation that women in sub-Saharan Africa face high HIV infection risk which continues during pregnancy. It is important to note that our data are from 2003–2006, before scale-up of antiretroviral therapy (ART). Limited recent data suggest that overall HIV incidence is declining in some high prevalence settings, likely as a result of the expansion of ART [39]. While there are no data specific to pregnant women, it is possible that incidence of HIV among pregnant women may also be declining.

While our analysis showed pregnant women had higher STI incidence rates compared to non-pregnant women, especially of chlamydia, pregnancy did not increase the risk of acquiring any of the infections in adjusted models. A previous analysis of these data, restricted only to women who had a pregnancy found that while sexual activity decreased during pregnancy, condom use was also lower among pregnant women [21]. Thus it may be that any increased risk in the crude analyses was caused by a change in condom use rather than a change in biological vulnerability. As noted, there are few data with which to compare these findings as there have been no previous studies examining increased risk of chlamydia, gonorrhea and trichomoniasis during pregnancy. Our findings showing that pregnancy did not appear to increase HIV incidence is in keeping with three previous analyses which also found no effect of pregnancy [17, 18, 40]. One study, conducted in Uganda, did find increased risk [19], however this finding has not been replicated.

This analysis has a number of strengths, as well as some limitations. As noted, there are very few data on STI incidence during pregnancy and only a small number of longitudinal studies have examined pregnancy as a risk factor for STI acquisition. Our analysis provides some of the first data on incidence of chlamydia, gonorrhea and trichomoniasis during pregnancy and provides new information regarding pregnancy as an STI risk factor. Another strength of our analysis was the methodological approach using a time-varying exposure and risk factors, including intravaginal practices which have not been included in previous analyses examining pregnancy as a risk factor for HIV acquisition.

A limitation of this analysis was reliance on self-report of sexual risk behaviors, including condom use which has been shown to be overestimated in biological validation studies [41]. It is also possible that there was misclassification of HC-use at non-pregnant visits which was also self-reported and may have had an impact on our results. Some data have shown that women using injectable progesterone-only hormonal contraceptives are at increased risk for STIs, including chlamydia, gonorrhea and HIV [32], however our findings do not support this. If injectable-HC use is associated with STI incidence and women underreported HC-use, this would have attenuated the effect of pregnancy on STI risk. Our analysis did not distinguish progesterone only hormonal contraceptives from combined injectable hormonal contraceptives. A previous analysis of these same data found a small increased risk of HIV among women using any injectable hormonal contraceptive which was non-significant and the results also did not support previous findings of greater risk from progesterone-only injectable hormonal contraceptives [42]. Another limitation is that we did not have data on STI status or risk behaviors collected directly from male partners, all data were based on women’s knowledge of male partner behaviors which may have been inaccurate or incomplete. Testing for bacterial STI was conducted using DNA PCR on urine samples which has been shown to have lower sensitivity compared to vaginal swabs for detecting infections, as well as lower specificity for detecting trichomonas [34, 35]. The PCR testing used in the study may have contributed to an underestimate of the prevalence of chlamydia and gonorrhea and an over estimate of trichomonas measured at enrollment and in detecting incidence during follow-up. It is important to note that while our measures of prevalence and incidence could have been affected by the lower sensitivity and specificity of urine PCR testing, the measurement error introduced would have been non-differential across pregnancy status and would bias our results toward the null for the main objective of comparing incidence between pregnant and non-pregnant women. A related additional limitation is that in 2004 Zimbabwe approved intermittent malaria preventive treatment for pregnant women pregnancy (IPTp) using sulfadoxine–pyrimethamine (SP) [43]. Sulfanomides, from which sulfadoxine is derived, have been used historically for the treatment of bacterial and parasitic STIs that include chlamydia, gonorrhea, and trichomonas [44]. It is therefore possible that the observed incidence of STI among women in Zimbabwe in the MIRA trial who became pregnant after 2004 and received SP may have been lower than would be found in an untreated population and this could have led to an underestimate of the relationship between pregnancy and STI incidence. Finally, our statistical analysis included a multilevel exposure variable (pregnancy and HC use status) which led to multiple comparisons which could have somewhat increased the likelihood of a type 1 error in finding statistical significance.

This analysis provides important information about women’s risk of acquiring sexually transmitted infections, including HIV. Overall we did not find that pregnancy increased the risk for acquiring chlamydia, gonorrhea, trichomoniasis or HIV.
